# The role of thrombomodulin lectin-like domain in inflammation

**DOI:** 10.1186/1423-0127-19-34

**Published:** 2012-03-27

**Authors:** Yi-Heng Li, Cheng-Hsiang Kuo, Guey-Yueh Shi, Hua-Lin Wu

**Affiliations:** 1 Department of Internal Medicine, National Cheng Kung University Hospital and College of Medicine, Tainan, Taiwan; 2 Cardiovascular Research Center, National Cheng Kung University, Tainan, Taiwan; 3 Department of Biochemistry and Molecular Biology, National Cheng Kung University, College of Medicine, Tainan 701, Taiwan; 4 Center for Bioscience and Biotechnology, National Cheng Kung University, Tainan, Taiwan

**Keywords:** Thrombomodulin, Lectin, Inflammation

## Abstract

Thrombomodulin (TM) is a cell surface glycoprotein which is widely expressed in a variety of cell types. It is a cofactor for thrombin binding that mediates protein C activation and inhibits thrombin activity. In addition to its anticoagulant activity, recent evidence has revealed that TM, especially its lectin-like domain, has potent anti-inflammatory function through a variety of molecular mechanisms. The lectin-like domain of TM plays an important role in suppressing inflammation independent of the TM anticoagulant activity. This article makes an extensive review of the role of TM in inflammation. The molecular targets of TM lectin-like domain have also been elucidated. Recombinant TM protein, especially the TM lectin-like domain may play a promising role in the management of sepsis, glomerulonephritis and arthritis. These data demonstrated the potential therapeutic role of TM in the treatment of inflammatory diseases.

## Review

### Introduction

**Figure 1:**
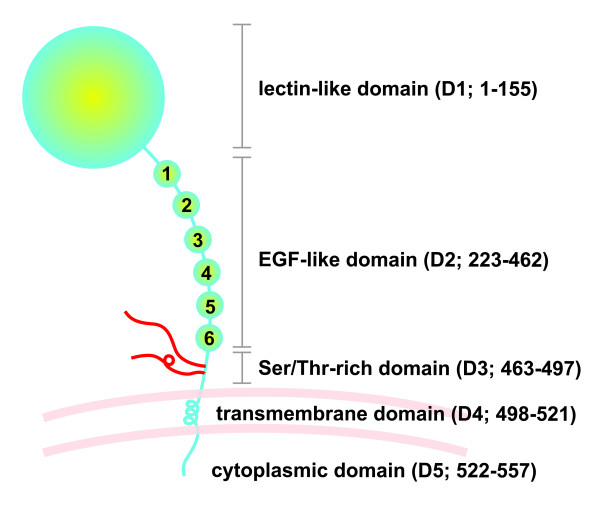
**Schematic presentation of structural domains of TM with corresponding sequence of amino acid**. EGF, epidermal growth factor. Ser, serine; Thr, threonine; D1, domain 1; D2, domain 2; D3, domain 3; D4, domain 4; D5, domain 5.

Thrombomodulin (TM) is a cell surface-expressed transmembrane glycoprotein which is originally identified on vascular endothelium. The cDNA sequence of TM has been determined with the cloning and sequencing of the human TM gene [1]. The mature human TM protein and its secondary structure have also been resolved [2]. TM protein has 557 amino acids, and its structure consists of 5 domains including a highly charged N-terminal lectin-like domain (D1), a domain with six epidermal growth factor (EGF)-like structures (D2), a serine and threonine-rich domain (D3), a transmembrane domain (D4) and a cytoplasmic domain (D5) [2] (Figure 1). TM on vascular endothelial cells is an important molecule in human natural anticoagulation system. After a stimulus, blood coagulation cascade amplifies and produces a high level of thrombin, the key effector of coagulation cascade. Natural anticoagulant mechanisms are activated to prevent excessive thrombin generation. TM acts as a thrombin receptor on the surface of vascular endothelial cells. The binding of TM to thrombin significantly decreases the thrombin's effect in conversion of fibrinogen to fibrin, and activation of coagulation factor V, VIII and platelet. Thrombin-TM complex catalyzes the activation of protein C about 1000 times faster than free thrombin. Activated protein C proteolytically inactivates the coagulation cofactor Va and VIIIa, thereby inhibiting the amplification of the coagulation system [3-5]. The importance of TM in natural anticoagulant system was demonstrated by the observation that transgenic mice with endothelium-specific loss of TM developed severe spontaneous thrombosis in the arterial and venous circulation, and inevitably led to the death of animal [6]. In addition to endothelium, TM is expressed in smooth muscle cell [7], platelet [8], monocyte [9], and cardiomyocyte [10]. TM is also expressed in some cancer cells and influences the growth and metastasis of cancer [11,12]. The presence of TM in these cells implies that the biological function of TM is not limited to anticoagulation [13]. Functionally, the region including the fourth, fifth, and sixth EGF-like structures of the second domain of TM (TMD2) is responsible for thrombin binding and protein C activation [14]. The lectin-like domain (the first domain of TM, TMD1) plays no role in the TM's anticoagulant activity. Although initial studies consider TM to be an anticoagulant, recent studies have revealed that TM, especially the TMD1, can modulate inflammatory process and has potent anti-inflammatory activity.

### TM and inflammation

Initially, TM is considered to have indirect anti-inflammatory activity and works mainly through its effect in producing activated protein C and suppressing thrombin activity. First, thrombin-TM complex produces a large amount of activated protein C which has a variety of anti-inflammatory activities. Activated protein C prevents inflammation-induced vascular permeability [15,16], suppresses inflammatory cytokine elevation in sepsis [17], inhibits leukocyte adhesion and decreases leukocyte chemotaxis [18]. After binding to endothelial protein C receptor (EPCR), activated protein C activates the protease-activated receptor 1 (PAR-1) and its downstream sphingosine-1 phosphate receptor 1 signaling pathway to execute the anti-inflammatory effects [15]. Second, TM decreases the pro-inflammatory effects of thrombin when TM binds to thrombin. Thrombin is a potent stimulus of inflammatory reaction. It disrupts the endothelial cell junction and increases tumor necrosis factor alpha production from monocytes [19]. It facilitates the recruitment of circulating monocytes by increasing endothelial expression of monocyte chemoattractant protein-1 (MCP-1), intercellular adhesion molecule-1 (ICAM-1) and vascular cell adhesion molecule-1 (VCAM-1) [20,21]. The thrombin signaling pathway is also via PAR-1 activation, but its downstream effector is coupled to the sphingosine-1 phosphate receptor 3. TM inhibits the interaction of thrombin with PAR-1, and decreasing thrombin's all pro-inflammatory effects. So TM plays a pivotal role in regulating the balance between activated protein C/EPCR/PAR1/sphingosine-1 phosphate receptor 1 and thrombin/PAR1/sphingosine-1 phosphate receptor 3 pathways during inflammation [22]. Through its thrombin inhibitory effect, infusion of recombinant D2 plus D3 of TM (rTMD23) protein could significantly reduce inflammation and decrease atherosclerosis formation in apolipoprotein E-deficient mice [23]. Third, thrombin-TM complex activates thrombin activatable fibrinolysis inhibitor (TAFI), also known as procarboxypeptidase R, a procarboxypeptidase that degrades several pro-inflammatory mediators such as bradykinin and complement factors C3a and C5a. Complement system is one of the important effectors in human immunity. Excessive activation of the complement system leads to several inflammatory diseases. Similar to the activation of protein C, TAFI is activated by the thrombin-TM complex with a catalytic efficiency of 1000-fold better than free thrombin alone. TAFI cleaves carboxyl terminal arginines of complement factors and bradykinin, inactivating their biological activities and downregulates the associated inflammatory reaction [24]. Although TMD1 does not involve in thrombin binding and has no effect in protein C activation, recent studies showed that TMD1 itself has direct anti-inflammatory activity.

### TMD1 structure

TMD1 consists of the first 1-155 amino acid residues in the N-terminal region of TM. There is a homology between TMD1 and the C-type lectin family. Originally, the C-type lectin was used to describe a group of Ca^2+^-dependent carbohydrate-binding (lectin) proteins. Subsequent studies demonstrated that the carbohydrate binding sites of C-type lectin usually exist in a compact protein region that became known as the C-type lectin domain and can be present in many other proteins. Previous study showed that TMD1 folds into a globular structure that consists of two alpha helices and six beta-strands forming two antiparallel beta-sheets [25,26]. Although TMD1 displays the essential features of the C-type lectin modules, it lacks traditional calcium binding site. Electron microscopy study demonstrated that TM is an elongated molecule about 20 nm long and TMD1 is a 5 nm nodular structure at its end [26]. These structure studies show that TM is a single chain membrane protein with TMD1 being furthest away from the plasma membrane. The location of TMD1 provides an ideal site for effective interaction with other molecules or cells. Interestingly, the structure and location of TMD1 in TM protein are similar to a group of protein currently known as C-type lectin receptors [27]. These receptors are transmembrane proteins bearing a C-type lectin domain with or without intracellular signal motifs. Some of the C-type lectin receptors may bind to pathogen through its C-type lectin domain and transduce signaling pathways into the cell to elicit inflammatory responses and play a critical role in host defense [27].

### Anti-inflammatory effect of TMD1

The anti-inflammatory effect of TMD1 was first demonstrated by observing that the transgenic mice with deleted TMD1 of TM protein (TM^LeD/LeD^) elaborated more inflammatory cytokines, including tumor necrosis factor and interleukin-1, and presented stronger inflammatory reaction after lipopolysaccharide (LPS) stimulation [28]. The transgenic mice have more leukocytes accumulation in the lungs after inhalation of gram-negative bacteria and increased mortality in endotoxin-induced sepsis. In vitro study showed that, in endothelial cells isolated from the TM^LeD/LeD ^mice, there was enhanced expression of ICAM-1 and VCAM-1, and increased neutrophils adhesion to endothelium [28]. Recombinant TMD1 (rTMD1) reduces the neutrophils adhesion to endothelium and suppresses activation of nuclear factor kappa B and mitogen-activated protein kinase pathways. TMD1 deletion does not interfere with the activation of protein C, indicating the direct anti-inflammatory effect of TMD1. The other clue showing TMD1 might be involved in inflammation comes from the observation that TMD1 plays an important role in maintaining cell-cell adhesion [29]. The A2058 melanoma cells transfected with wild type TM clustered closely together with strong cell-cell adhesion. However, in A2058 cells transfected with TMD1-deleted TM, the cells were dispersed as single cells in nonconfluent cell densities as parental A2058 cells. Antibody against lectin-like domain of TM was able to block cell-cell contacts and inhibited close clustering morphology in the wild type TM-transfected cells [29]. Taken together, these results provide a possibility that TM may maintain the integrity of endothelial junctions, thereby keeping a quiescent state of blood vessels [30].

**Figure 2:**
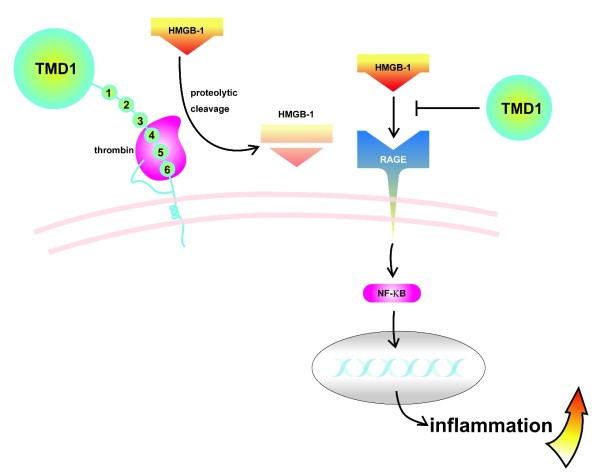
**TM suppresses HMGB1-mediated inflammation**. HMGB1 mediated-pro-inflammatory pathways are inhibited by TM through two mechanisms. First, Soluble TMD1 sequesters HMGB1, and results in decreasing its binding to RAGE, thereby suppressing the inflammatory response. Second, thrombin-TM complex cleaves HMGB1, resulting in generation of inactive HMGB1 fragment. HMGB1, high mobility group B1; RAGE, receptor for advanced glycation end products; TMD1, TM domain 1.

The definite molecular mechanisms mediating the anti-inflammatory effect of TMD1 has been elucidated recently and at least 2 binding targets of TMD1 have been identified. The first one is high mobility group box 1 (HMGB1) protein. HMGB1 is released from necrotic cells [31] or secreted by inflammatory cells such as activated monocytes [32] after cytokine stimulation. It is a potent pro-inflammatory mediator and, recently, becomes a target of treatment for inflammation [33]. HMGB1 binds to its endothelial cell surface receptor, receptor for advanced glycation end products (RAGE) or toll-like receptors to trigger the activation of downstream pro-inflammatory pathway and tissue damage. TM binds to HMGB1 protein through its D1 domain and interferes the binding of HMGB1 to RAGE [34]. The prevention of HMGB1 to bind RAGE by TMD1 decreases the pro-inflammatory effect of HMGB1 and subsequent inflammation (Figure 2). Later study shows that, after binding to TMD1, HMGB1 can be effectively degraded by thrombin-TM complex to a less pro-inflammatory form and the inflammatory reaction is further down-regulated [35].

**Figure 3:**
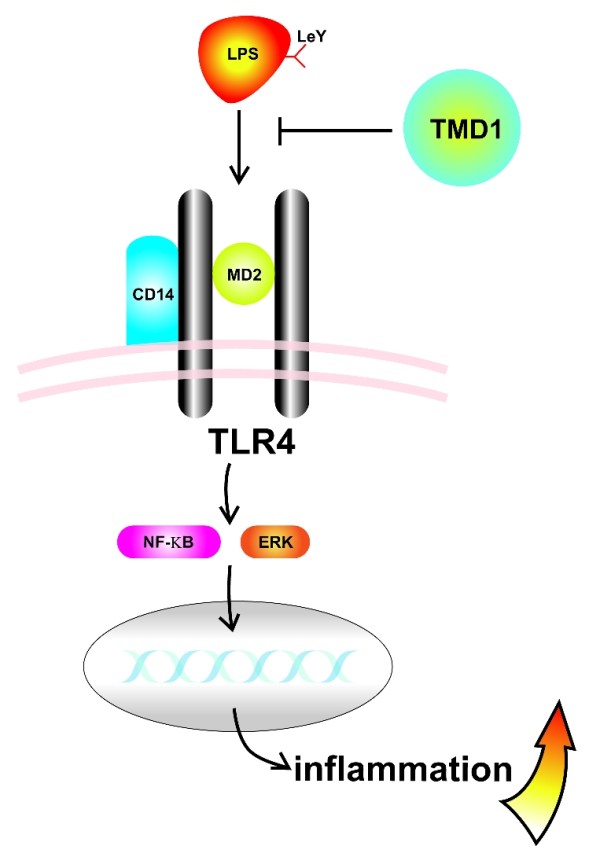
**TMD1 interferes with LPS-mediated inflammation**. Soluble TMD1 binds to Lewis Y antigen on the LPS, thereby preventing engagement of LPS, CD14, and TLR4, resulting in reduced activity of NFκB and mitogen activated kinase/ERK pathways. LeY, Lewis Y antigen; TLR4, toll-like receptor 4; NFκB, nuclear factor κB; ERK, extracellular signal regulated protein kinases.

Another binding target of TMD1 is the carbohydrate Lewis Y antigen in LPS on gram-negative bacteria [36]. LPS is a major cell wall component of gram-negative bacteria and acts as a major endotoxin that elicits strong inflammatory responses during infection. LPS interacts with CD14 and toll-like receptor on the cell surface and transduces signals from the cell membrane into the cytosol, starting the downstream inflammatory signaling pathway [37]. Previous study demonstrated that LPS of *Helicobacter pylori *contains sequences related to Lewis X and Lewis Y antigens and interacts with selectins [38]. Soluble TMD1 or rTMD1 could directly bind to LPS, block the interaction of LPS with CD14 and reduce the subsequent LPS-induced inflammatory reaction by suppressing the activation of mitogen-activated protein kinase and nuclear factor kappa B signaling pathways. The release of pro-inflammatory cytokines and expression of inducible nitric oxide synthase were also decreased [36]. The Lewis Y antigen in LPS is the specific molecular target that is bound by TMD1 (Figure 3). By binding to the Lewis Y antigen, rTMD1 can specifically induce the agglutination of *Klebsiella pneumoniae *in the presence of Ca^+2 ^and enhance the phagocytosis of the bacteria by macrophages [36]. These phenomena indicated that TMD1 may function as a natural opsonin for innate immunity against gram-negative bacteria.

Finally, the anti-inflammatory activity of TMD1 may also relate to its ability to suppress the activation of complement system directly. Increased complement activation was found in the TM^LeD/LeD ^mice [39]. TM downregulates the alternative pathway of complement activation by directly enhancing the endogenous complement inhibitors, complement factor I and H, to inactivate C3b [40]. Several TM genetic mutations in the TMD1 causing defects in TM binding of complement factor H and C3b were observed in patients with atypical hemolytic-uremic syndrome, a disease with complement overactivation [40]. However, the definite mechanism of molecular interaction of TM with complement system needs further investigation.

### Recombinant TM for inflammatory diseases

#### TM and sepsis

All the previous data demonstrate that the anti-inflammatory activity of recombinant TM protein, especially the rTMD1, has therapeutic potential in inflammatory diseases. Sepsis is a clinical syndrome caused by bacterial or viral infection-induced systemic inflammatory response [41]. Endotoxin or LPS of the infectious pathogen is responsible for pathophysiological events occurring during sepsis and leads to systemic inflammation and coagulation. Disseminated intravascular coagulation (DIC), which is manifested as coagulation abnormalities, pulmonary vascular permeability dysfunction, and acute respiratory distress syndrome (ARDS) are common complications of sepsis and lead to high mortality [42]. Recombinant TM has been investigated in several animal models of sepsis. In rats, pretreatment of recombinant TM protein containing all the extracellular domains (rTMD123) significantly reduced the mortality of LPS-induced sepsis [43]. rTMD123 treatment decreased inflammatory cells infiltration in lung and liver. It decreased the elevation of tumor necrosis factor-alpha, interleukin-6 and HMGB1. Furthermore, *in vitro *study showed that rTMD123 administration inhibited the nuclear factor-kappa B activation by blocking I kappa B phosphorylation in macrophages [43]. In mouse sepsis model, mice receiving rTMD1 injection have significantly less tumor necrosis factor-alpha elevation and inflammatory cells infiltration in lung tissue. rTMD1 treatment decreased sepsis mortality caused by LPS and gram-negative bacteria [36]. All the data suggest that recombinant TM protein can be used to treat inflammation in sepsis. Saito et al. used rTMD123 to perform a phase III clinical trial in patients with DIC caused by hematological malignancy or infection [44]. They compared the DIC resolution rate between the patients received rTMD123 and heparin. rTMD123 treatment resulted in a better DIC resolution than heparin. The decrease of plasma thrombin-antithrombin complex and D-dimer levels were significantly greater in rTMD123 group. However, the overall mortality was similar between the 2 groups [44]. Up to now, there has no data available on administration of rTMD1 to patients with sepsis. Because rTMD1 has no effect in binding thrombin and activating protein C, the bleeding risk after injection of rTMD1 should be less than rTMD123. Further clinical trials are necessary to evaluate the rTMD1 effect in patients with sepsis.

#### TM and glomerulonephritis

Glomerulonephritis is one of the major causes of renal failure. The disease results from glomeruli injury caused by a variable of insults, such as infection, immunological disorders and vasculitis, and the resultant inflammation and coagulation lead to glomeruli damage and renal failure. In a rat model of glomerulonephritis induced by simultaneous administration of LPS and anti-glomerular basement membrane antibody, Ikeguchi et al. found rTMD123 injection could decrease the thrombus deposition and inflammatory cells infiltration in the glomeruli, salvage the acute renal dysfunction and reduce the mortality of the animals [45]. The complement activation and glomerular C3 deposition are major manifestations in this animal glomerulonephritis model. They further found the plasma level of TAFI was greatly increased after TM injection, and TAFI inhibitor significantly diminished the inhibitory effect of TM on leukocyte infiltration [45].

Acute kidney injury resulting from renal ischemia is another important cause of renal failure. Inflammation, renal tubular epithelial cell dysfunction and apoptosis play important roles in the pathogenesis of ischemic renal injury. Ozaki et al. created an ischemia/reperfusion renal injury model in rats by performing right nephrectomy and left renal artery clamping. Intrarenal injection of rTMD123 could preserve a better renal function after ischemia/reperfusion renal injury [46]. The severity of renal tubular damage was evaluated by the extent of tubular dilatation, degeneration, and cast formation. Compared with saline injection, rTMD123 could decrease renal tubular damage and inflammatory cells infiltration. Furthermore, in vivo study showed that rTMD123 treatment reduced the apoptosis of renal tubular epithelial cells after ischemia/reperfusion renal injury; while in vitro study showed rTMD123 treatment could reduce hydrogen peroxide-induced endothelial cells apoptosis [46]. The protective effect of TM in ischemic renal injury was reconfirmed in another animal model. Sharfuddin et al. performed suprarenal aorta clamping to reduce 90% aortic flow for 60 min and induced ischemia/reperfusion renal injury [47]. In this model, pretreatment of recombinant TM protein containing D1 and D2 (rTMD12) significantly reduced the severity of renal tubular damage, inflammatory cells infiltration and mortality in rats. Interestingly, in this study, a point mutation in the TMD2 region which is responsible for protein C activation was generated. They found the mutant rTMD12 has no ability to activate protein C but still has the same renoprotective effect as the wild type TM indicating the importance of TMD1 anti-inflammatory effect [47]. Our study demonstrated LPS injection alone could induce severe glomerulonephritis in mice. Mice receiving only rTMD1 injection had less extent of glomerulonephritis and better renal function than the control mice [36]. These data reconfirmed that the TMD1 has direct anti-inflammatory function that is independent of activated protein C effect. In addition, activation of epidermal growth factor receptor (EGFR) was demonstrated in glomerular disease, especially rapidly progressive glomerulonephritis [48,49]. Our recent study showed that rTMD1 interfere EGFR signaling through interaction with LeY, thereby suppressing EGF-mediated angiogenesis and tumor growth [50]. Taken together, rTMD1 is promising in the treatment of EGFR-mediated inflammation, including glomerulonephritis.

#### TM and arthritis

Rheumatoid arthritis is a chronic inflammatory autoimmune disease of joints. Pathological examination revealed chronic synovial inflammation and progressive destruction of the affected joints. The major inflammatory cell type in synovial tissue that is responsible for pro-inflammatory cytokines production is macrophage. Tumor necrosis factor-alpha and interleukin-1 from macrophage play a pivotal role in the pathogenesis and inflammatory progression of rheumatoid arthritis. Previous studies showed that the soluble TM level is increased in the urine and joint fluid from the patients with rheumatoid arthritis [51,52] and TM is synthesized and expressed by the synovial lining cells [9,52]. These data implied that TM might play important role in the pathogenesis of rheumatoid arthritis. TM^LeD/LeD ^mice developed more severe inflammatory arthritis in an anti-collagen antibody-induced arthritis model [39]. The mice developed more significant swelling in the paws and inflammatory cells infiltration in the synovial tissues than the wild type counterparts. Treatment of the mice with rTMD1 significantly improved the arthritis severity, decreased the thickness of synovial lining, suppressed the macrophage infiltration in synovial tissue, and reduced the HMGB1 expression in these macrophages [39]. There was increased complement deposition in the joint. Further in vitro studies showed that rTMD1 could suppress the complement pathways activation [39]. In addition, our current study suggested that rTMD1 may protect against arthritis progression through sequestration of LeY, which was increased in synovial fluid from patients with rheumatoid arthritis [53]. These data implied that rTMD1 may be used as a therapeutic agent in the inflammatory arthritis.

## Conclusion

Inflammation is a complex pathological process that is induced by various mediators secreted from inflammatory cells. Coagulation cascade is one of the systems that involved in the inflammatory process. Although traditionally known as an anticoagulant protein, TM has been identified to play an important role in modulating inflammation through several indirect and direct pathways [54]. The molecular switch of TM from anticoagulation to regulation of inflammation may be triggered by inflammatory stimulation thus exposing its lectin-like domain for further interaction and signal transduction [55]. The direct anti-inflammatory effect of TMD1 has been found recently and its molecular targets have also been elucidated. The use of recombinant TM protein to treat inflammatory diseases in human and animal studies has demonstrated TM to be a new potential therapy for patients with sepsis, glomerulonephritis and arthritis. Because TMD1 does not participate in anticoagulation, rTMD1 should not have the bleeding side effect. It is promising in the treatment of inflammatory diseases. Further clinical trials will be necessary to elucidate its safety and clinical usefulness.

## Abbreviations

ARDS: Acute respiratory distress syndrome; DIC: Disseminated intravascular coagulation; EGF: Epidermal growth factor; EPCR: Endothelial protein C receptor; HMGB1: High mobility group box 1; ICAM-1: Intercellular adhesion molecule-1; LPS: Lipopolysaccharide; MCP-1: Monocyte chemoattractant protein-1; PAR-1: Protease-activated receptor 1; RAGE: Receptor for advanced glycation end products; TAFI: Thrombin activatable fibrinolysis inhibitor; TM: Thrombomodulin; TMD1: Thrombomodulin domain 1; TMD12: Thrombomodulin domain 1 plus 2; TMD23: Thrombomodulin domain 2 plus 3; TMD123: Thrombomodulin domain 1 2, plus 3; VCAM-1: Vascular cell adhesion molecule-1.

## Competing interests

The authors declare that they have no competing interests.

## Authors' contributions

YHL and CHK were involved in drafting the manuscript. GYS and HLW designed the concept and made revision of the manuscript. All authors read and approved the final manuscript.
